# Prevalence and characterization of quinolone-resistant *Escherichia coli* isolated from retail raw beef and poultry meat in Egypt

**DOI:** 10.5455/javar.2023.j702

**Published:** 2023-09-30

**Authors:** Dina Ibrahim, Amal Awad, Gamal Younis

**Affiliations:** Department of Bacteriology, Mycology and Immunology, Faculty of Veterinary Medicine, Mansoura University, Mansoura, Egypt

**Keywords:** Escherichia coli, retail poultry meat, beef, quinolone-resistant determinants, PMQR

## Abstract

**Objective:**

The goal of this study was to look at quinolone-resistant (QR) *Escherichia coli* (*E. coli*) from retail beef and poultry meat in Egypt by looking at the QR mechanisms in the resistant strains.

**Materials and Methods:**

In total, 120 samples of raw poultry meat (*n* = 60) and beef meat (*n* = 60) were purchased from Mansoura retail stores between January and March 2021, and evaluated microbiologically for *E. coli*. Then, an antimicrobial sensitivity test was applied to all isolates. The prevalence of QR *E. coli *with concern for the QR determinants, including quinolone resistance-determining regions (QRDRs) mutations, the plasmid-mediated quinolone resistance gene (PMQR), and the efflux pump activity were determined.

**Results:**

The total prevalence of *E. coli* was 34.2% (41/120). Noticeably, the prevalence of *E. coli* in poultry meat (40%, 24/60) was higher than that of beef (28%, 17/60). All strains were assessed for their antimicrobial susceptibility using the disc diffusion technique; the highest rate of resistance (100%) was displayed to clindamycin and cefuroxime, followed by ampicillin (97.6%), doxycycline (92.7%), amoxicillin-clavulanate (92.7%), nalidixic acid (NA) (80.5%), sulfamethoxazole/trimethoprim (70.7%), chloramphenicol (63.4%), gentamicin, and azithromycin (58.5% each). Multiple antimicrobial resistance (strains resistant to three or more antimicrobial classes) was displayed by 97.6% of *E. coli* isolates. Regarding QR, 37 isolates could resist at least one of the examined quinolones. Regarding PMQR genes, *qnrS* was determined in 70% (7/10) of QR *E. coli,* while *qnr*A, *qnrB, *and *qnr*D were not identified. While the mutations determined regions of QR in the resistant *E. coli *isolates, S83L was the most prevalent in gyrase subunit A either alone or combined with D87N and D87Y, and three isolates of QR *E. coli* isolates revealed a *topoisomerase IV subunit* mutation harboring S80I. 20% of the isolates displayed efflux activity, as NA showed a considerable difference between its zones of inhibition.

**Conclusion:**

The high prevalence of antimicrobial-resistant *E. coli,* with concern for QR strains harboring different resistance mechanisms in poultry meat and beef, threatens the public’s health. Thus, standard manufacturing procedures and adequate hygiene conditions must be followed in all phases of meat preparation, production, and consumption, and public knowledge should be improved.

## Introduction

Antibiotics are routinely used in intensive farming systems, resulting in gene reservoirs for antimicrobial resistance that could transfer to other hosts or the environment. As a matter of truth, antibiotics are routinely used in an unsustainable way as precautionary preventative procedures, for mass therapy without a precise diagnosis, or for infections that can be avoided [1]. Antimicrobial resistance develops in pathogenic and commensal microorganisms due to their use in food-producing animals [2]. Horizontal gene transfer can transfer resistance genes carried on mobile genetic materials to the human flora during the human body’s transit or colonization [3,4]. As a result, the human flora, containing microorganisms that are possibly hazardous to humans, such as nosocomial pathogens, may become resistant [5]. Furthermore, given the tremendous selection pressure of continual antimicrobial medication use, new [multidrug-resistant (MDR)] variants may colonize animals. Prospective variants could be highly virulent and better suited to humans, creating a public health risk [6].

The gastrointestinal system, in particular, is a hotspot for horizontal gene transfer between species and within species. *Escherichia coli* and *Enterococcus* spp., for example, are well-known inhabitants of the mammalian gastrointestinal tract and have been demonstrated to be effective suppliers and recipients of antibiotic resistance determinants [7]. As a result, these resistance genes constitute an indirect threat.

Resistance determinants are usually present on portable genetic materials, as well as the high bacterial load in the intestine is advantageous for genetic translocation; hence, food is likely to have a role in commensal bacteria resistance transmission. Transmission can happen through the exposure or ingestion of tainted foods, which can expose the public and food handlers. Furthermore, food handlers may act as reservoirs, resulting in widespread foodborne outbreaks. The extent to which individuals are subjected to foods infected with antimicrobial-resistant bacteria is determined by various factors that might increase or decrease the bacterial load, as well as hygiene precautions implemented during food processing, shipping, and preparation. Antimicrobial-resistant bacteria can be transmitted through surfaces, hands, equipment, and so on, from one food to another [8].

The fluoroquinolone antimicrobial agent class has gained widespread acceptability among patients in hospitals and those residing in the community, and its use seems to be on the rise [9]. Because they directly block DNA synthesis, fluoroquinolones (and prior quinolones) are unique among antibacterial drugs in clinical usage. The medicine appears to inhibit DNA gyrase and topoisomerase IV by binding with combinations containing DNA and one of the two specific enzymes. During the topoisomerization phase, fluoroquinolones appear to trap the enzyme on DNA, producing a physical barrier to replication fork [10], RNA polymerases in addition to helicase movement in the transcription process [11].

Resistance to quinolones is caused by three mechanisms: drug-targeting mutations, drug-accumulation mutations, and plasmids that shield cells from the toxic impacts of quinolones [12]. fluoroquinolones´ resistance in Gram-negative bacteria is linked to a decrease in porins and drug accumulation. However, measures of diffusion rates imply that porin reductions alone are not enough for resistance to show [13].

Bacteria, which resist different antimicrobials and pass naturally from vertebrates into people, also can pose human illness and are a direct threat to human health. In Egypt, there is limited information on quinolone-resistant (QR)* E. coli* recovered from various food samples. Because of their dissemination as opportunistic pathogens, the constant rise in the presence rate of QR *E. coli* isolates is especially concerning and demonstrates the importance of expanding our research of their origins, reservoirs, and transmission routes. Thus, this study aimed to investigate the prevalence of QR *E. coli *in retail beef and poultry meat in retail stores located in Mansoura, Egypt. Moreover, for determination of QR determinants in the resistant *E. coli* isolates.

## Materials and Methods

### Samples collection

In total, 120 samples of meat consisting of retail poultry meat (*n* = 60) and fresh raw beef (*n* = 60) were collected from retail stores located in Mansoura, Egypt between February and April 2021. These samples were gathered and labeled in tightly closed plastic bags and transferred in an ice box under aseptic conditions to the Bacteriology, Mycology, and Immunology Department Laboratory, Faculty of Veterinary Medicine at Mansoura University, Mansoura to further bacteriological investigations.

### Isolation and identification

About 25 gm of every sample were suspended in 225 ml of Tryptone Soya Broth (TSB; Oxoid, UK) and incubated for 18–24 h at 37°C. A loopful from each pre-enriched broth was streaked on Eosin methylene blue (EMB) medium (Oxoid) and then incubated for 24 h at 37°C. Presumed colonies (green with metallic shin) were picked up and purified on Tryptone Soya Agar (TSA; Oxoid, UK) and biochemically characterized according to MacWilliams [14]. Subsequently, retrieved *E. coli* isolates were confirmed by PCR targeting *16S rRNA*.

### Molecular identification of E. coli isolates 

Genomic DNA was extracted following Ramadan et al. [15]. In brief, three to five* E. coli* colonies were inoculated into TSB and then incubated for 18 h at 37°C. One milliliter of that overnight bacterial culture was separated using centrifugation for 2 min at 10,000×*g*. The sediment was cleared with DNA/RNA-free water, homogenized, and then boiled for 15 min at 95°C. The supernatants from boiled lysates were employed as DNA templates. The recovered DNA templates were adjusted to a concentration of 100 ng/l using a Nanodrop (Nanodrop 1000, Thermo Scientific, UK). PCR directed at the region of 16S ribosomal RNA was done to confirm *E. coli*, and a uniplex PCR assay was conducted using the following primer: F: GACCTCGGTTTAGTTCACAGA and R: CACACGCTGACGCTGACCA using the cyclic condition illustrated in Table 1 as described previously by the referred author [16].

### Antimicrobial susceptibility test

To determine antibiotic resistance phenotypic profiles, the Clinical and Laboratory Standards Institute’s (CLSI) instructions for the diffusion technique were followed [17]. Antimicrobial susceptibility tests were conducted using such antimicrobial discs (Bioanalyze/Turkey): amoxicillin-clavulanate (AMC, 30 µg), cefuroxime (CXM; 30 µg), ampicillin (AM; 10 µg), chloramphenicol (C; 30 µg), gentamicin (CN; 10 µg), doxycycline (DO; 30 µg), clindamycin (DA; 2 µg), azithromycin (AZM; 15 µg), sulfamethoxazole/trimethoprim (SXT; 25 µg), nalidixic acid (NA; 30 µg), norfloxacin (NOR; 10 µg), ciprofloxacin (CIP; 5 µg), and levofloxacin (LEV; 5 µg). *Escherichia coli *ATCC 25922 was used in the study as a quality assurance*. *MDR *E. coli *isolates exhibit resistance to over three distinct antibiotic classes [18]. In addition, the MAR index was calculated using the approach given by Osundiya et al. [19], which involves the antibiotics number that an isolate showed resistance (a) divided by the total antibiotics number utilized in this research (b). The following is the calculation formula: MAR index = a/b.

### Detection for the determining regions of the QR

QR was assessed through DNA gyrase subunit A (*gyr*A) as well as topoisomerase IV subunit C (*par*C) mutation analysis of the quinolones’ resistance determining regions (QRDR). The *gyr*A and *par*C genes in the areas that determine QR were amplified using PCR. For the *gyr*A gene, the forward primer’s sequence: 5¢-TAC ACC GGT CAA CAT TGA GG-3¢ and the reverse primer: 5¢-TTA ATG ATT GCC GCC GTC GG-3¢ were used. The ampliﬁcation was performed in a 96-well Applied Biosystem, 2720 thermal cycler as follows: (i) a first denaturation phase lasting 4 min at 94°C, then 30 cycles of denaturation lasting 1 min at 92°C, annealing of 1 min at 64°C, and extension for 2 min at 74°C; then finally, (ii) a last extension process lasting 10 min at 74°C [20]. For *par*C, a PCR protocol for amplification of *par*C following Weigel et al. [21] was conducted using the following primers pair [22]: 5¢-AAA CCT GTT CAG CGC CGC ATT-3¢ and 5¢- GTG GTG CCG TTA AGC AAA-3¢ with an initial denaturation of 94°C lasting for 4 min, then 30 cycles of, denaturation lasting 1 min, annealing lasting 30 sec at 55°C, extension lasting 45 sec at 72°C, and lastly a final cycle running 10 min at 72°C. Amplification results were seen using an electrophorized agarose gel stained with ethidium bromide to check the gene fragment’s sizes. The puriﬁed products of PCR from both genes were subsequently subjected to sequencing.

### Analysis of QRDRs

QIA quick PCR product extraction kit from Qiagen Inc. in Valencia, California, was utilized for the refinement of the PCR products. Cycle sequencing kit with Bigdye Terminator V3.1, cat-number 4336817, from the Perkin-Elmer in Foster city, California, and Applied Biosystems 3130 genetic analyzer (HITACHI, Japan) were used to do gene sequencing and analysis. In addition, kit number CS-901 of 100 reactions was utilized to purify the sequence reaction, as well as Applied Biosystems 3130 automated DNA sequencer (ABI, 3,130, United States of America). Through the National Center for Biotechnology Information website (http://www.ncbi.nlm.nih.gov/BLAST), the Basic Local Alignment Search Tool was applied to verify the nucleotide sequences with those in the GenBank database.

**Table 1.** List of oligonucleotide primers used in this study.

Gene	Primer name	Primer sequence (5¢→3¢)	(bp)	Reference
16S rRNA	16S-F	F: GACCTCGGTTTAGTTCACAGA	585	[16]
	16S-R	R: CACACGCTGACGCTGACCA		
DNA *gyrA*	gyrA-F	F: TACACCGGTCAACATTGAGG	648	[20]
	gyrA-R	R: TTAATGATTGCCGCCGTCGG		
Topoisomerase IV subunit C	Par C-F	F: AAACCTGTTCAGCGCCGCATT	395	[22]
	Par C-R	R: GTGGTGCCGTTAAGCAAA		
PMQR	qnrS-F	F: ACGACATTCGTCAACTGCAA	417	[25]
	qnrS-R	R: TAAATTGGCACCCTGTAGGC		
	QnrA-F	F: TCAGCAAGAGGATTTCTCA	627	[23]
	QnrA_R	R: GGCAGCACTATTACTCCCA		
	QnrB-F	F: CGACCTGAGCGGCACTGAAT	515	[24]
	QnrB-R	R: TGAGCAACGATGCCTGGTAG		
	QnrD-F	F:CGAGATCAATTTACGGGGAATA	582	[26]
	QnrD-R	R: AACAAGCTGAAGCGCCTG		

### Detection for plasmid-mediated quinolone resistance (PMQR) gene 

*Escherichia coli *isolates were submitted to PCR for determination of PMQR, including *qnr*A [23], *qnr*B [24], *qnr*S [25], and *qnr*D [26] using specific primers (Metabion international/AG/Germany) shown in Table 1, and a thermal cycler condition mentioned by the referred authors was used to amplify the specific fragments. PCR products were separated by electrophoresis on a gel of agarose 1% having 0.5 mg/l of ethidium bromide and then photographed under UV light with a Gel Documentation System (Cleaver Scientific Ltd, UK).

### Assessment of antibiotic sensitivity test in the availability of the efﬂux pump inhibitor 

The antibiotic sensitivity testing of QR isolates was done in the existence and absence of the inhibitor of the efﬂux pump, epinephrine [27]. The broth dilution technique was employed to identify the minimal inhibitory dose of epinephrine for the isolates under research, following the CLSI guidelines [28]. At 37°C, two tubes of Muller Hinton (MH) broth (Oxoid) and MH broth containing 150 g/ml of epinephrine (MISR CO.-EGYPT) were inoculated with each strain for 24 h. On MH agar plates, a loopful of each tube was spread, antibiotic discs were inserted, and the plates were incubated at 37°C overnight. A ruler was used to measure the inhibition zones encircling the antibiotic discs. The distinction in the inhibition zones was evaluated in the existence and absence of epinephrine [29].

## Results

### Prevalence of E. coli isolates

**Figure 1.**
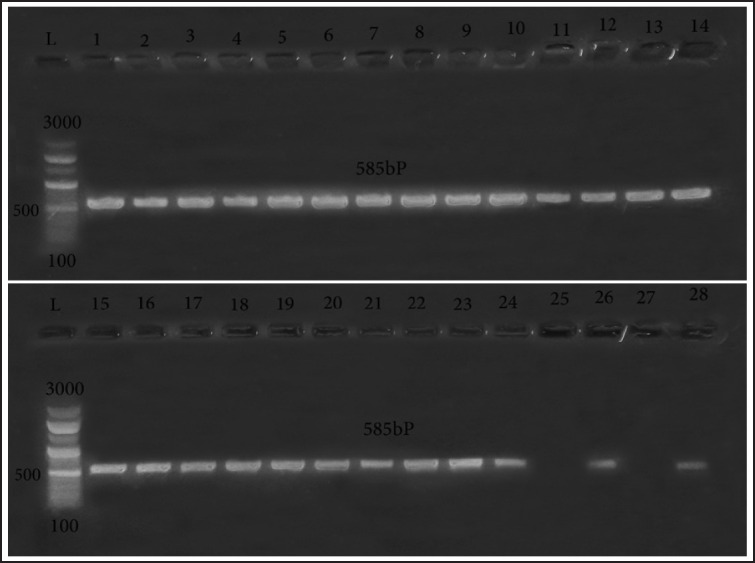
*Escherichia coli* identification targeting 16S rRNA (585 bp). Lane L: 100:3,000 bp DNA size marker, Lane 1:24, 26, 28: positive samples. Lane 25, 27: negative samples.

In this work, 120 samples were examined for the existence of *E. coli* using the conventional cultural techniques stated to evaluate the prevalence of *E. coli* from poultry meat (*n* = 60) and beef meat (*n* = 60). Forty-one isolates (34.2%) were biochemically revealed to be *E. coli* from poultry meat (24/60; 40%) and beef meat samples (17/60; 28.33%). All 41 biochemically identified isolates were then confirmed as *E. coli* by PCR (Figure 1).

### Antibiotic susceptibility testing

The tested isolates exhibited a remarkable resistance to DA and CXM (41/41, 100%), followed by AM (40/41, 97.6%), DO (38/41, 92.7%), AMC (38/41, 92.7%), SXT (29/41, 70.7%), and C (26/41, 63.4%), CN and AZM (24/41, 58.5% each). Regarding resistance to quinolones, 80.5% (33/41) of *E. coli* isolates had resistance to NA, followed by CIP (20/41, 48.78%), LEV (18/41, 43.9%), and NOR (16/41, 39%) (Table 2). *Escherichia coli* isolates exhibiting resistance to three or more different antimicrobial classes were termed MAR. In accordance with the prior terminology, MAR was detected in 97.6% (40/41) of the tested isolates (Table 3).

### Detection of QRDRs

**Figure 2.**
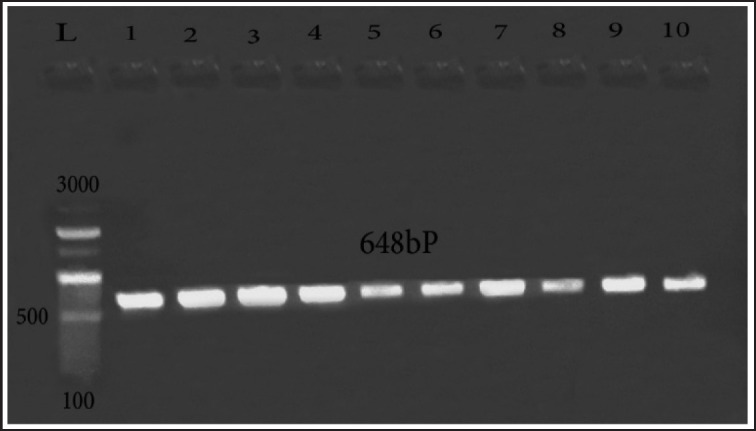
Amplification of *gyr*A at 648-bp in the QRDRs in QR *E. coli* strains. Lane L: 100:3,000-bp DNA size marker, Lane 1:10 QR *E. coli* strains.

**Figure 3.**
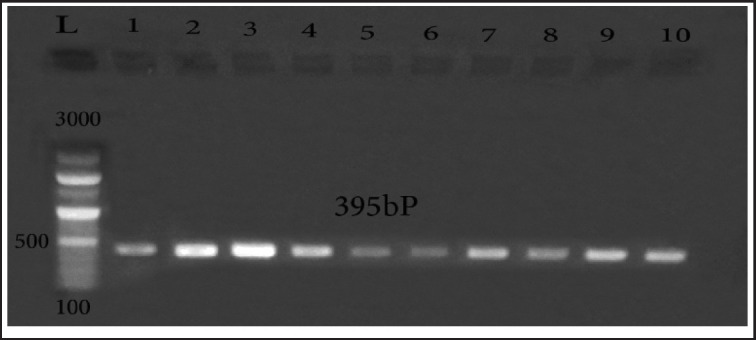
Amplification of *par*C at (395-bp) in the QRDRs. Lane L: 100:3,000-bp DNA size marker, Lane 1:10 QR *E. coli* strains.

Ten QR isolates (isolates displayed resistance to all quinolone antimicrobials used) were investigated for mutations in gyrA of QRDR (Figure 2) and *par*C (Figure 3) by PCR and then DNA sequencing. The results of sequencing revealed that 5 out of the 10 isolates held S83L mutation in *gyr*A; among them, 2 isolates harbored D87N and D87Y alterations at the same gene of *gyr*A at 83 and 87 residues (S83L/D87N/D87Y). The obtained sequences for mutations in DNA *gyr*A were submitted to GenBank under the accession numbers OM105873, OM105874, OM105875, OM105876, and OM105877. For *par*C, one typed mutation at the *par*C gene coding Ser80I was found in three isolates, and they were uploaded in the GenBank under the following accession numbers: OM105878, OM105880, and OM105882.

### Determination of PMQR genes

**Figure 4.**
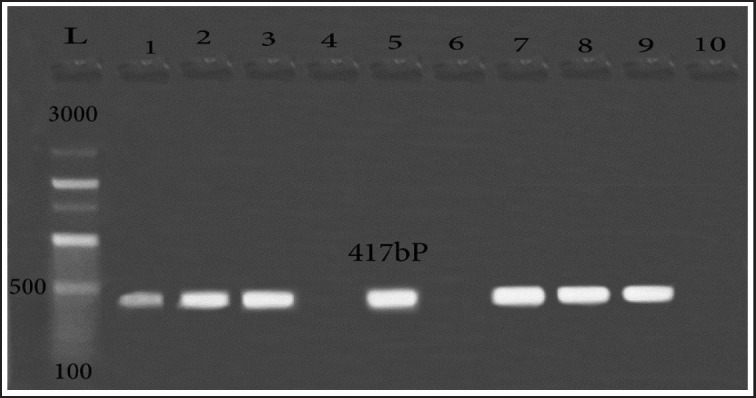
Amplification of PMQR (*qnr*S, 417-bp) in QR *E. coli* strains. Lane L: 100:3,000-bp DNA size marker, Lane 1,2,3,5,7,8,9: positive samples. Lane 4,6,10: negative samples.

The PMQR was evaluated to explore further resistance’s mechanisms in QR *E. coli* isolates. The *qnr*s gene encoding QR was observed in 7 isolates out of the 10 resistant isolates (Figure 4). While, *qnr*A, *qnr*B, and *qnr*D were not determined in the examined isolates.

### The inhibitor of the efﬂux pump (epinephrine) influence on the antibiograms

Epinephrine, as an inhibitor of the efﬂux pump, was employed to evaluate the action of efﬂux for 10 QR isolates. The minimal inhibitory concentration (MIC) of epinephrine was found to be 500 µg/ml. Lower doses for the inhibitor of the efflux pump (150 µg/ml) than inhibitory ones were used. All *E. coli* strains that appeared to a specific antibiotic resistance were checked. Sensitivity tests for antibiotics were run with and without epinephrine, measuring the distinction in the inhibition zones (Abp—Ab 0) with availability or lack of epinephrine. The considerable discrepancy was 2 mm at least. (2 of 10 samples) for NA showed considerable distinctions in the zones of inhibition suggesting that it was possibly efﬂuxed out of the cells. While 98% of the tested isolates did not show the difference in the inhibition zone (with and without the addition of epinephrine) with NA. The disparity in the inhibition zones of NOR, CIP, and LEV for all the tested isolates was determined to be insigniﬁcant, indicating that no role to the efflux pump in the resistance of these isolates.

**Table 2.** Antimicrobial susceptibility testing results.

Antibiotics	Family	Disc code	CPD	Chicken meat isolates (*n* = 24)		Beef meat isolates (*n* = 17)		Total (*n* = 41)	
				Resistant	Sensitive	Resistant	Sensitive	Resistant	Sensitive
AM	β-lactam	AM	10	24 (100%)	0 (0.00%)	16 (94%)	1 (6%)	40 (97.6%)	1 (2.4%)
AMC	Β-lactams	AMC	30	24 (100%)	0 (0.00%)	14 (82.4%)	3 (17.6%)	38 (92.7%)	3 (7.3%)
CN	Aminoglycoside	CN	10	18 (75%)	6 (25%)	6 (35.3%)	11 (65.7%)	24 (58.5%)	17 (41.5%)
DO	Tetracycline	DO	30	23 (95.8%)	1(4.2%)	15 (88.2%)	2 (11.8%)	38 (92.7%)	3 (7.3%)
AZM	Macrolide	AZM	15	16 (66.7)	8 (33.3%)	8 (47%)	9 (53%)	24 (58.5%)	17 (41.5%)
C	Phenicols	C	30	22 (91.7%)	2 (8.3%)	4 (23.5%)	13 (76.5%)	26 (63.4%)	15 (36.6%)
DA	Lincosamide	DA	2	24 (100%)	0 (0.00%)	17 (100%)	0 (0.00%)	41 (100%)	0 (0.00)
SXT	Sulphonamide	SXT	25	23 (95.8%)	1 (4.2%)	6 (35.3%)	11 (65.7%)	29 (70.7%)	12 (29.3%)
CXM	Cephalosporin	CXM	30	24 (100%)	0 (0.00%)	17 (100%)	0 (0.00%)	41 (100%)	0 (0.00%)
NA	Quinolones	NA	30	23 (95.8%)	1 (4.2%)	10 (58.8%)	7 (41.2%)	33(80.5%)	8 (19.5%)
NOR	Fluoroquinolone	NOR	10	12 (50%)	12 (50%)	4 (23.5%)	13 (76.5%)	16 (39%)	25 (61%)
CIP	Fluoroquinolone	CIP	5	13 (54.2%)	11 (45.8%)	7 (41%)	10 (59%)	20 (48.78%)	21 (51.22%)
LEV	Fluoroquinolone	LEV	5	10 (41.7%)	14 (58.3%)	8 (47%)	9 (53%)	18 (43.9%)	23 (56.1%)

## Discussion

The spreading of Qs resistance in *E. coli* has considerably increased recently as a result of MDR phenotypes commonly emerge in this organism [30,31]. Accordingly, the already high health risk posed by these strains as food-chain intermediaries for antibiotic-resistance genes is greatly increased. In this study, a total of 120 poultry meat and beef samples were investigated for *E. coli *contamination. *Escherichia coli* prevalence in the examined samples was 34.2% (41/120). Noticeably, the contamination rate (40%, 24/60) of poultry meat was higher than that of beef samples (28.3%, 17/60). In agreement with our findings, Moawad et al. [32] and Belotindos et al. [33] have recorded a high rate of contamination of poultry meat compared with beef. While, Adzitey et al. [34] recorded an overall prevalence rate of 55% of *E. coli* from both poultry meat and beef, while, beef samples were reported to be more contaminated (80%) than poultry samples (20%).

Antibiotics are frequently administered to chickens during the raising process, both for disease prevention and treatment and for body development [35]. Penicillins, sulfonamides, tetracyclines, and quinolones are the most often utilized antibiotic classes in bred poultry [36]. Consequently, in this research, the greatest resistance frequency was detected against DA and CXM with a percentage of 100% followed by AM (97.6%), and then DO and AMC with a percentage of 92.7%, and then NA, SXT, and C with percentages of 80.5%, 70.7%, and 63.4%, respectively. In addition, 58.5% of the tested strains were resistant to CN and AZM. These findings are concerning and directly threaten human health. Antimicrobial resistance has the potential to minimize the effectiveness of first-trial zoonosis treatment and constrict postdiagnosis therapeutic options. Resistant (foodborne) bacteria strains are more likely than susceptible types to produce invasive sickness, greater mortality, and hospitalization [37].

Fluoroquinolones are synthesized drugs with a broad range of action that are frequently applied for treating bacterial infections [38]. As a result of their widespread use, fluoroquinolones resistance has emerged, raising the possibility of treatment failure [39]. In this study, 90.2% (37/41) of QR *E. coli* isolated were detected from both sources. Twenty-three *E. coli* isolates out of 24 isolates from poultry samples were resistant to quinolones (95.8%), while, 82% (14/17 isolates) of beef isolates showed QR which goes in line with Caruso et al. [1] and Belotindos et al. [33]. The highest resistance against the quinolone group was recorded against NA (80.5%) followed by CIP (48.78%), LEV (43.9%), and then NOR (39%).

**Table 3.** Pattern of antimicrobial susceptibility testing.

Pattern	Resistance pattern	Isolates no.(%) *N* = 41	MAR index
1	AMC, AM, DO, AZM, C, SXT, CXM, CN, DA, NA	4(10%)	0.77
2	AMC, AM, DO, AZM, C, SXT, CXM, CN, DA, NA, NOR	1(2.4%)	0.85
3	AMC, AM, DO, AZM, C, SXT, CXM, CN, DA, NA, NOR, LEV	1(2.4%)	0.92
4	AMC, AM, DO, AZM, C, SXT, CXM, CN, DA, NA, CIP, LEV	1(2.4%)	0.92
5	AMC, AM, DO, AZM, C, SXT, CXM, CN, DA, NA, NOR, CIP, LEV	6(15%)	1
6	AMC, AM, DO, C, SXT, CXM, CN, DA, NA	2(5%)	0.69
7	AMC, AM, DO, C, SXT, CXM, CN, DA, NA, CIP	1(2.4%)	0.77
8	AMC, AM, DO, C, SXT, CXM, CN, DA, NA, NOR, CIP, LEV	1(2.4%)	0.92
9	AMC, AM, DO, C, SXT, CXM, AZM, DA, NA, NOR, CIP, LEV	2(5%)	0.92
10	AMC, AM, DO, C, CXM, AZM, CN, DA, NA	1 (2.4%)	0.69
11	AMC, AM, DO, C, CXM, AZM, CN, DA, NA, CIP	1(2.4%)	0.77
12	AMC, AM, DO, SXT, CXM, AZM, DA, NA	1(2.4%)	0.6
13	AMC, AM, DO, SXT, CXM, AZM, DA, NA, NOR, CIP	1(2.4%)	0.77
14	AMC, AM, DO, SXT, CXM, C, DA, NA	1(2.4%)	0.6
15	AMC, AM, DO, SXT, CXM, C, DA, NA,NOR, CIP	1(2.4%)	0.77
16	AMC, AM, SXT, CXM, C, AZM, DA, NA,NOR,CIP,LEV	1(2.4%)	0.85
17	AMC, AM, DO, CXM, AZM, CN, DA, LEV	1(2.4%)	0.6
18	AMC, AM, DO, CXM, AZM, CN, DA,NOR,CIP, LEV	1(2.4%)	0.77
19	AMC, AM, DO, CXM, SXT, CN, DA, NA	1 (2.4%)	0.6
20	AMC, AM, DO, CXM, AZM,C, DA, NA, CIP	1(2.4%)	0.69
21	AMC, AM, DO, CXM, SXT, DA, NA	1(2.4%)	0.54
22	AMC, AM, DO, CXM, SXT, DA, NA, CIP	1(2.4%)	0.6
23	AMC, AM, DO, CXM, SXT, DA, NA, NOR, LEV	1(2.4%)	0.69
24	AMC, AM, DO, CXM,CN, DA, NA, LEV	1(2.4%)	0.6
25	AMC, AM, DO, CXM,CN, DA, CIP, LEV	1(2.4%)	0.6
26	AM, DO, CXM, DA, NA	1(2.4%)	0.38
27	AM, AMC, DO, CXM, DA, CIP, LEV	1(2.4%)	0.54
28	AMC, AM, C, SXT, CXM, DA.	1 (2.4%)	0.46
29	AMC, DO, AZM, CXM, DA	1 (2.4%)	0.38
30	AM, DO, CXM, DA	1 (2.4%)	0.31
31	CXM, DA	1 (2.4%)	0.15

Alterations in the genes encoding the relevant enzymes are known to be the most frequent mechanism of Qs resistance. Genetic analysis of selected *E. coli* isolates relied on the *gyr*A and *par*C genes’ sequences revealed that 5 strains carried a point mutation at *gyr*A with a serine to leucine change at position 83, one of the widely recorded resistance granting mutations [40]. In addition, other *gyr*A mutations were detected at position 87 with an aspartate to asparagine substitution and an aspartate to tyrosine substitution. Single-or double-point alterations at *gyr*A were recorded in this work. Regarding *par*C of QRDR, a serine into isoleucine substitution at position 80 was detected in 3 isolates. The combination between both single and double *gyr*A mutations with *par*C mutations has been recorded. Similarly, prior researches report that the most frequent forms of known amino acid change in *E. coli *were in *gyr*A (S83 L and D87 N) as well as *par*C (S80I) [40–42].

The majority of the genes that cause antibacterial resistance are found on plasmids and other portable genetic components, which can and frequently disseminate to bacteria of various genera and species. [43,44]. In this research, most of the Qs-resistant isolates (7/10) were PMQR-positive (*qnr*S). Similarly, previous reports worldwide have reported a high prevalence of PMQR-positive among Q-resistant *E. coli* [45–48] which highlights their significant contribution to acquiring Qs resistance. On the other hand, a lower prevalence of PMQRs was mentioned among QR *E. coli* in Egypt (26.6%) [49], Taiwan (14.9%) [50], and Germany (3.7%) [51]. According to Woyda et al. [52], chicken production isolates were more likely than human clinical isolates to carry acquired AMR genes (65.7%), and they also carried an average of more acquired AMR genes per isolate. Furthermore, Juraschek et al. [51] verified that the existence of *qnr* alone can result in phenotypic (*fluoro*) QR without the need for PMQR or point mutations in the relevant chromosomal area.

Efflux pumps, also known as MDR pumps, have been identified as a major determinant of antibiotic concentration within a bacterial cell. As a result, inhibiting efflux pumps can be used to increase the concentration of antibiotics inside a pathogenic cell, thereby increasing the efficacy of these drugs [53]. In the present study, epinephrine was used as an EPI, by investigating its effect in certain concentrations (150 mg/ml) on the inhibition zone values of selected antibiotics. 20% of the isolates revealed the efflux action indicating that this drug was probably efﬂuxed from the cells, while, 80% of the tested isolates did not show that action with NA. NOR, CIP, and LEV showed no differences in their inhibition zones for all tested isolates, indicating that no role to the efflux pump action in the resistance. Similarly, a lower prevalence of efflux pump effect was recorded by Hooper and Jacoby [54] (12.1%), Hamed et al. [55] (18.3%), and Vieira et al. [56] among quinolone resistant (QR) isolates suggested a higher contribution of other resistance mechanisms. Porin reductions and decreased bacterial drug accumulation are linked to fluoroquinolone resistance in Gram-negative bacteria, although assessments of diffusion levels indicate that reductions of porin alone, typically are not enough to cause resistance [13,57,58].

In this study, 97.6% of the tested isolates expressed MDR phenotypes with a MAR index of more than 0.2. The highest resistance rate was recorded in 32% of these tested isolates that resisted 9 various antibiotic classes and 20% of *E. coli* isolates resisted 8 different antibiotic classes. While, 21%, 12%, and 10% of tested isolates displayed resistance to 7, 6, and 5 different antibiotic classes, respectively. The lowest percentage of isolates (2.5%) resist to two and four different antibiotic classes. These findings agree with those of recent investigations [33, 58–60] demonstrating greater incidence of MDR between Q-resistant *E. coli*. The high percentages of resistant strains isolated in this study may pose a direct risk to consumers by colonizing their intestinal tract until conditions are favorable for extraintestinal infection, or indirectly by transferring resistance genes to human commensal flora [61]. The MAR index is a reliable, valid, and affordable method for tracing the origins of antibiotic-resistant organisms [62]. A MAR of more than 0.2 indicates a great threat of contamination origin in areas where antimicrobials are routinely utilized [63]. In this investigation, the highest MDR was presented in *E. coli* with a MAR value of 1.00, in 17% of the tested isolates which goes in line with Ayandele et al. [64] who recorded a high MDR with a MAR index of 1.00 in 34% of *E. coli* isolates and Jaja et al. [65] who reported MAR indexes ranged from 0.2 to 0.5. Antimicrobial drugs used in veterinary medicine must be used responsibly and prudently to preserve both animal and human health [66]. To address the antimicrobial resistance crisis, various degrees of safety precautions must be considered such as "tertiary prevention" (improving the immune systems ability of animals to react to diseases) [67] and vaccines based on broadly preserved antigens [68,69].

## Conclusion

In conclusion, personal protective precautions, such as the wearing of safety clothes such as gloves and masks, as well as the usage of basic health measures such as hand washing and showering, should be promoted to avoid the dissemination of resistant microbes from food of animal origins to humans. Furthermore, slaughterhouses in addition to food handling practices must be considered in the effort to reduce foodborne transmission, in accordance with the concept of “farm-to-fork”.

## List of Abbreviations

AM, Ampicillin; AMC, Amoxicillin-clavulanate; AZM, Azithromycin; C, Chloramphenicol; CIP, Ciprofloxacin; CLSI, Clinical and Laboratory Standards Institute’s; CN, Gentamicin; CXM, Cefuroxime; DA, Clindamycin; DO, Doxycycline;*gyr*A, DNA gyrase subunit; EPI, efflux pump inhibitor; LEV, Levofloxacin; MAR, multiple antibiotic resistance; MDR, Multidrug-resistant; MH, Muller Hinton; NA, Nalidixic acid; NOR, Norfloxacin; *par*C, Topoisomerase IV subunit; PCR, polymerase chain reaction; PMQR, Plasmid-mediated quinolone resistance gene; QIA, Qiagen; QR, Quinolone resistant; QRDRs, Quinolone resistance-determining regions; TSA, Tryptone Soya Agar; TSB, Tryptone Soya Broth; SXT, Sulfamethoxazole/trimethoprim.

## Availability of data and materials

The corresponding author will supply the datasets produced and/or analyzed in the present research upon reasonable request. Sequence data for *gyr*A and *par*C genes available in the GenBank repository under the dissertation title: Studies on the mechanisms of quinolones resistance in *E. coli* isolated from retail poultry and meat.
